# Crosslinking-controlled biopolymer biomaterials for tissue engineering and regenerative medicine: network chemistry, processing, and mechanistically informed validation

**DOI:** 10.3389/fbioe.2026.1962375

**Published:** 2026-10-22

**Authors:** Stefano Bellucci

**Affiliations:** 1 Qi S.r.l., Pomezia, Italy; 2 Universidad Ecotec, Samborondón, Ecuador

**Keywords:** biofabrication, biological validation, biopolymer biomaterials, crosslinking, hydrogels, mechanobiology, regenerative medicine, tissue engineering

## Abstract

Crosslinking is often treated as a technical step used to stabilize biopolymer scaffolds, yet in regenerative biomaterials it is a primary design variable that simultaneously controls network mechanics, viscoelasticity, swelling, molecular transport, degradation, ligand accessibility, and cell–material interactions. This Review examines crosslinked biopolymer biomaterials for tissue engineering and regenerative medicine through that network-centered perspective. Collagen and gelatin, hyaluronic acid, silk fibroin, chitosan, alginate, fibrin, and related matrices are considered with emphasis on how covalent, photoinduced, enzymatic, ionic, physical, and dynamic covalent crosslinking generate distinct biological microenvironments. We analyze how crosslinking is coupled to processing routes including extrusion bioprinting, electrospinning, freeze-drying, injectable hydrogel formation, and porous-scaffold fabrication, and why the timing of network formation can be as important as nominal polymer composition. We propose a mechanistically informed evidence chain connecting material definition to hydrated-state properties, cellular response, *ex vivo* or *in vivo* behavior, and tissue-specific regenerative outcomes. Particular attention is given to quantitative network characterization, reproducibility, sterilization, storage, aging-related property drift, and controls needed to distinguish genuine network effects from formulation confounders. Clinical trials, patient data, patent landscapes, regulatory pathways, commercialization, and product-development strategies are intentionally excluded. Crosslinking should be designed and reported as a multidimensional regulator of the regenerative microenvironment rather than as a single measure of scaffold stability.

## 1 Introduction

Biopolymer biomaterials occupy a distinctive position in tissue engineering because they can combine biochemical recognition with hydrated, remodelable network structures. Collagen, gelatin, hyaluronic acid (HA), silk fibroin (SF), chitosan, alginate, fibrin, and cellulose-derived matrices have all been processed into hydrogels, porous scaffolds, fibers, films, meshes, injectable depots, and bioinks. Their appeal is often summarized in terms of biocompatibility and resemblance to extracellular matrix (ECM), but this description is incomplete. A regenerative construct is not defined by polymer identity alone. Cells experience a time-dependent three-dimensional network whose density, connectivity, ligand presentation, pore structure, water content, degradation rate, and viscoelastic response are strongly shaped by crosslinking chemistry and processing history (Lee and Mooney, 2001; Lutolf and Hubbell, 2005; Catoira et al., 2019). This distinction matters because biological responses are sensitive to the mechanical and temporal characteristics of the matrix. The classic observation that matrix elasticity can influence stem-cell lineage decisions established stiffness as an instructive cue (Engler et al., 2006). Subsequent work showed that elastic modulus alone is not sufficient: stress relaxation, remodeling, spatial heterogeneity, and time-dependent mechanics can change spreading, differentiation, and matrix production even at similar initial stiffness (Chaudhuri et al., 2016). Crosslinking is therefore not merely a method for making a soft polymer “stronger”. It changes the set of forces and transport processes available to cells, as well as the rate at which a construct evolves after implantation. Recent biomaterial studies increasingly exploit this coupling. Dynamic HA networks can combine injectability with secondary covalent stabilization and immunomodulatory bone repair (Guo et al., 2024); enzymatically crosslinked collagen type II/HA hydrogels can provide a biomimetic delivery environment for cartilage cells (Torabi Rahvar et al., 2024); and GelMA or SF-based matrices are being engineered to couple crosslink density to degradation, oxidative-stress control, multicompartment architecture, and tissue-specific function (Han et al., 2025; Wang et al., 2025; Natarajan et al., 2026). These examples point to a broader design principle: the regenerative outcome is determined by a network state, not by a material label. The present Review focuses on crosslinked biopolymer biomaterials as experimentally rigorous regenerative platforms. In accordance with the scope of the Research Topic, the discussion is restricted to *in vitro*, *ex vivo*, and *in vivo* preclinical evidence; clinical studies, patient data, case reports, and clinical trials are outside scope. Hybrid systems are included only when the biopolymer remains the central matrix and the added phase clarifies how mechanics, transport, bioactivity, or cell–material interactions are modified. The objective is not to catalogue every hydrogel formulation. Instead, we ask which crosslinking and processing variables must be measured, how they influence biological interpretation, and what evidence is required to support claims of material-guided regeneration.

## 2 Scope and conceptual framework

The scope is deliberately preclinical and materials-mechanistic. Clinical trials, patient data, case reports, patent landscapes, regulatory pathways, commercialization, and product-development strategies are not reviewed. This boundary is intended to keep the analysis focused on how network chemistry, processing, characterization, and biological models support mechanistic claims before clinical translation.

This is a critical narrative Review rather than a systematic review. Literature was selected to represent major biopolymer classes, established crosslinking mechanisms, advanced processing routes, and recent studies in which material properties were connected to biologically meaningful preclinical outcomes. Crosslinking and printing approaches have also been reviewed as interdependent variables in biopolymer-based tissue engineering (Patrocinio et al., 2023). Foundational studies are used where they define concepts that remain essential, while recent work from 2024 to 2026 is emphasized to illustrate current directions in dynamic networks, biofabrication, immune-aware design, and rigorous *in vivo* validation. The organizing framework is a causal sequence: polymer and crosslinker chemistry determine network architecture; network architecture determines hydrated-state mechanics, stress relaxation, swelling, transport, degradation, and ligand accessibility; these properties shape cell adhesion, cytoskeletal organization, differentiation, immune response, and matrix remodeling; and those cellular processes ultimately influence tissue formation and host integration. Figure 1 summarizes this cascade. The value of the framework is that it discourages attribution of a biological effect to the entire formulation when the responsible variable may be a change in crosslink density, pore structure, degradation products, or transport. Four principles guide the discussion. First, crosslink density should be treated as a measured or inferable material variable rather than a nominal recipe parameter. Second, properties should be characterized in the hydrated state and, where possible, under conditions that approximate physiological exposure. Third, processing and sterilization are part of network design because they can change chemistry and microstructure. Fourth, biological validation should include controls that separate network effects from differences in polymer concentration, filler loading, surface chemistry, or soluble bioactive agents.

**FIGURE 1:**
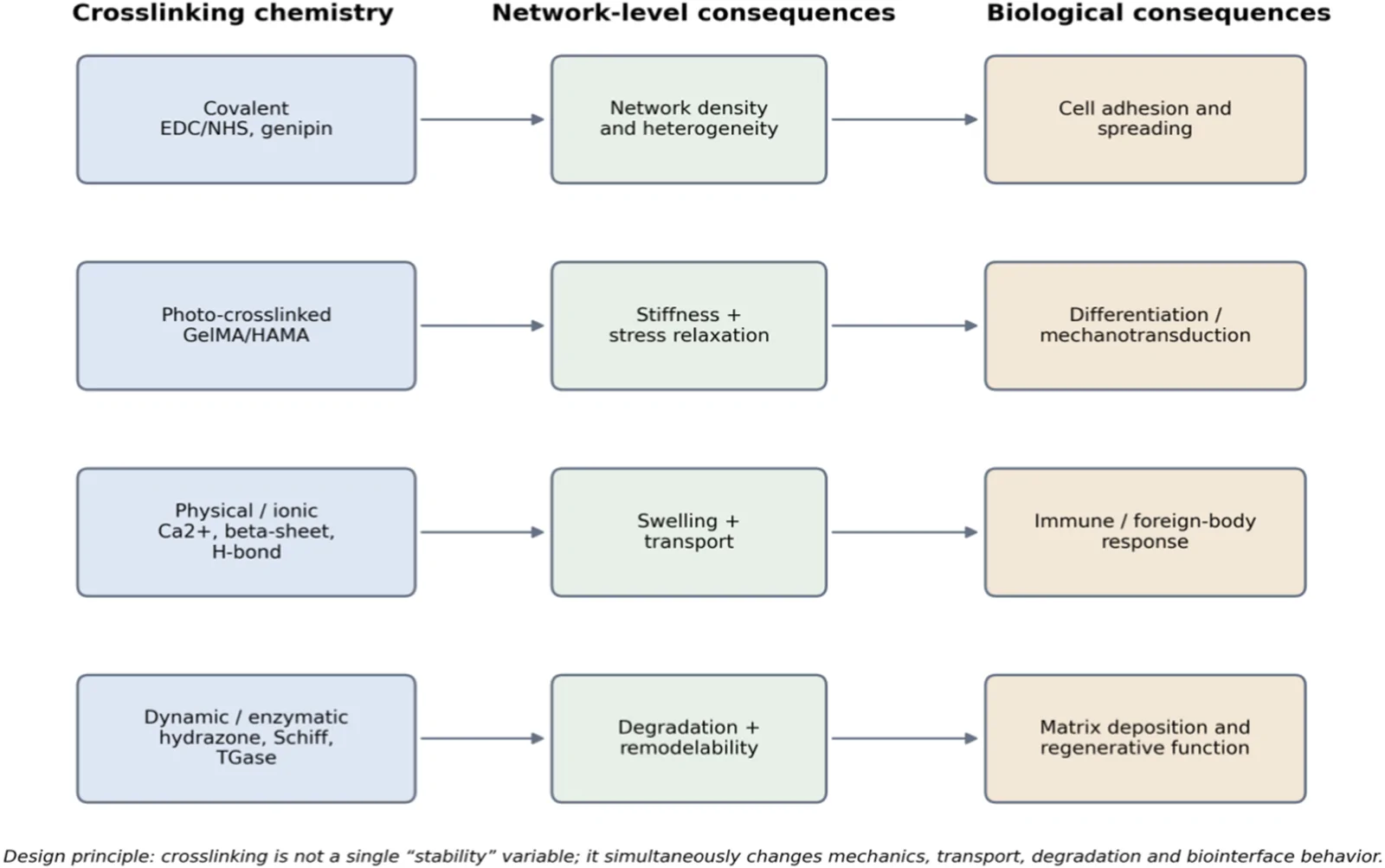
Crosslinking chemistry-to-biology cascade in biopolymer biomaterials. Covalent, photoinduced, physical/ionic, dynamic, and enzymatic network formation alter network density and heterogeneity, stiffness and stress relaxation, swelling and transport, and degradation/remodelability. These properties subsequently influence adhesion, mechanotransduction, immune response, matrix deposition, and regenerative function. Original schematic prepared for this Review. Bellucci | Crosslinking-Controlled Biopolymer Biomaterials | Frontiers in Bioengineering and Biotechnology v i ew In re.

## 3 Network chemistry as a master design variable

### 3.1 Covalent crosslinking: stability versus remodeling

Covalent crosslinking is widely used to stabilize protein and polysaccharide matrices. In collagen and gelatin systems, carbodiimide chemistry, genipin, glutaraldehyde alternatives, methacrylation, and other functionalization routes can increase resistance to dissolution and tune mechanics. The central tradeoff is that every additional junction reduces chain mobility and can alter accessibility of cell-binding or protease-sensitive motifs. For collagen, excessive chemical stabilization may preserve gross architecture while suppressing cell-mediated remodeling; for gelatin, the same increase in network density can improve shape retention but reduce swelling and diffusion. These competing effects are why crosslinker concentration alone is an inadequate descriptor (Shoulders et al., 2009; Van Vlierberghe, 2016). Covalent networks are particularly valuable when dimensional stability is a dominant requirement, but regenerative design rarely favors maximal crosslinking. The target is a window in which the scaffold remains intact long enough to support cell infiltration and matrix deposition, yet can still be degraded or reorganized at a rate compatible with tissue formation. This principle is evident in recent injectable and granular GelMA strategies, in which covalent photopolymerization is combined with architectures that retain local porosity or injectability rather than producing a homogeneous, permanently dense block (De Maeseneer et al., 2024).

The principal crosslinking classes, their strengths, confounders, and minimum reporting variables are compared in Table 1.

**TABLE 1:** Crosslinking strategies: advantages, limitations, and variables that should be reported.

Class	Examples	Strengths	Limitations/confounders	Minimum reporting
Permanent covalent	EDC/NHS, genipin, stable click reactions	High stability; broad chemical versatility	Can reduce ligand accessibility and remodeling	Functional-group ratio, reaction time, residual crosslinker, gel fraction, wet mechanics
Photoinduced	GelMA, HAMA, methacrylated SF	Spatial/temporal control; biofabrication-compatible	Light attenuation, radicals, oxygen inhibition, thickness gradients	Degree of substitution, photoinitiator, wavelength, irradiance/dose, thickness
Ionic/physical	Alginate–Ca2+, H-bonding, hydrophobic association, SF beta-sheets	Mild, often reversible	Media-dependent drift; batch sensitivity	Ion concentration, bath composition, secondary structure, exchange/stability tests
Dynamic covalent	Hydrazone, imine, boronate, thiol–aldehyde	Self-healing, shear-thinning, adaptive mechanics	Strong pH/ionic/competitor dependence	Bond chemistry, k_on/k_off or K_eq when available, gelation time, stress relaxation, exchange/dissolution conditions
Enzymatic	Transglutaminase, peroxidase-mediated systems	Mild aqueous conditions	Enzyme diffusion/activity gradients	Enzyme concentration/activity, reaction time, conversion, residual activity
Dual/interpenetrating	Dynamic + photo; ionic + covalent	Decouples injectability from long-term stability	Complexity can obscure causal variables	Each component, sequence/timing, individual controls, combined mechanics/degradation

### 3.2 Photo-crosslinking and methacrylated biopolymers

Photo-crosslinkable biopolymers such as GelMA and HAMA provide unusually direct control over crosslinking in space and time. They are compatible with molding, lithography, and bioprinting, and can be cured after deposition to preserve complex geometries. GelMA retains gelatin-derived cell-adhesive and matrix-metalloproteinase-sensitive motifs while adding methacryloyl groups that enable light-initiated network formation (Yue et al., 2015; Klotz et al., 2016). This combination has made GelMA a reference material for studying how network mechanics and architecture affect cell behavior. The advantage of optical control is accompanied by several confounders. Polymer degree of functionalization, photoinitiator chemistry, light wavelength, radiant dose, oxygen inhibition, sample thickness, and post-gelation pH can all change the final network. A recent Frontiers study showed that the autofluorescence lifetime of GelMA was sensitive to photocrosslinking conditions and post-gelation pH, illustrating that network state can drift after curing and that non-destructive monitoring may be valuable (Rodriguez and Quinn, 2026). Thus, reporting only polymer concentration and irradiation time does not fully define a photocrosslinked biomaterial. Photocrosslinking also interacts with biological payloads. In GelMA-based constructs for peripheral-nerve-related applications, mechanical reinforcement and antioxidant functionality were engineered while maintaining Schwann-cell compatibility (Han et al., 2025). In periodontal bone repair, GO/GelMA hydrogels combined a photocrosslinked protein-derived matrix with an inorganic nanophase and demonstrated antibacterial and osteogenic effects *in vitro* and *in vivo* (Cheng et al., 2026). Such studies are most informative when the contribution of the network is separated from that of the secondary component through matched controls.

### 3.3 Physical, ionic, and self-assembled networks

Physical crosslinking relies on non-covalent interactions such as ionic coordination, hydrogen bonding, hydrophobic association, beta-sheet formation, and crystallization. These networks often permit mild processing and cell encapsulation because they avoid highly reactive crosslinkers. Alginate is the canonical example: divalent cations create ionically crosslinked junction zones, producing rapid gelation under aqueous conditions (Lee and Mooney, 2012). Silk fibroin provides a contrasting protein example in which beta-sheet-rich domains can stabilize the network and strongly influence degradation. The reversibility of non-covalent interactions can be an advantage when injectability, stress dissipation, or cell-driven remodeling is required, but it also increases sensitivity to ionic exchange and physiological media. For alginate, calcium redistribution can alter local crosslink density after implantation; for silk, the fraction and distribution of beta-sheet domains influence both stiffness and enzymatic degradation. Zhu et al. (2025) directly examined how SF hydrogel degradation affects encapsulated stem-cell proliferation and chondrogenesis, reinforcing the point that degradation should be treated as an active biological variable rather than a late-stage materials test. Recent regenerative studies exploit ionic crosslinking in tissue-specific ways. PRP-loaded alginate hydrogels supported fibrocartilage-like matrix formation and meniscal repair in a rabbit model, with comparison to sham and hydrogel-only controls (Resmi et al., 2025). An injectable alginate–cell cross-linked gel was also used to promote cell orientation and tendon regeneration, illustrating how the geometry of cell–network interactions can convert a simple polysaccharide matrix into a mechanically instructive system (Yamaguchi et al., 2025).

### 3.4 Dynamic covalent and enzymatic networks

Dynamic covalent networks seek to reconcile two properties (Han et al., 2022) that are difficult to combine in permanently crosslinked hydrogels: mechanical integrity and local rearrangement. Hydrazone, imine/Schiff-base, boronate ester, thiol-aldehyde, and related reversible bonds can permit shear-thinning, self-healing, injectability, or on-demand dissolution. The relevant design variable is not simply whether a bond is “dynamic”, but its exchange rate under the pH, ionic strength, redox state, and competing nucleophile concentrations expected in the intended biological environment. A recent HA-based system for bone tissue engineering combined hydrazone-mediated dynamic crosslinking with secondary photopolymerization. The first network provided shear-thinning and self-healing, whereas the second increased mechanical stability; the construct supported osteogenic differentiation and macrophage M2 polarization and improved calvarial bone formation *in vivo* (Guo et al., 2024). This dual-network strategy exemplifies a general solution to the injectability–stability tradeoff. Other dynamic systems emphasize reversibility rather than permanent reinforcement. Thiol-aldehyde addition between functionalized HA and chondroitin sulfate produced an injectable, self-healing wound dressing that could be dissolved on demand, demonstrating that controlled network disassembly can be a design objective rather than a failure mode (Johnson et al., 2024). Enzymatic crosslinking provides another route to mild network formation. In collagen type II/HA hydrogels, enzymatic crosslinking was used to generate a cell-delivery matrix for cartilage engineering while preserving a biomimetic composition (Torabi Rahvar et al., 2024). Network dynamics can also be introduced through reversible physical reinforcement rather than bond exchange in the polymer backbone. Chalard et al. (2024) combined photocrosslinked GelMA with supramolecular GalC7 fibers that were grown within the hydrogel. The fibrous phase increased the initial Young’s modulus but progressively dissolved because it was maintained by non-covalent interactions, producing time-dependent softening. This example is useful because it separates two forms of stability within one construct: a persistent covalent GelMA network and a transient supramolecular reinforcement. For regenerative applications, such architectures suggest that the relevant design variable is not simply whether a material is crosslinked, but which network components persist, exchange, or disappear over the time scale of cell-driven remodeling. The main advantages, limitations, and minimum reporting variables associated with the major crosslinking classes are compared in Table 1.

## 4 Matrix-specific crosslinking sensitivities

### 4.1 Quantifying crosslink density, mesh size, and network dynamics

Crosslink density is not a single directly observable quantity. Chemical conversion measures the fraction of reactive groups consumed, whereas effective crosslink density refers to elastically active junctions and excludes loops, dangling chains, and other defects. Accordingly, nominal crosslinker concentration should not be used as a surrogate for network connectivity. Equilibrium swelling can be interpreted through Flory–Rehner-type treatments to estimate molecular weight between crosslinks and mesh size, but these estimates assume an idealized homogeneous network and are sensitive to polymer–solvent interaction parameters. Rheological or rubber-elasticity estimates use the plateau modulus to infer elastically active chain density, while gel fraction quantifies the insoluble fraction without uniquely specifying network topology. Spectroscopic methods such as NMR, FTIR, or Raman can quantify functional-group conversion but likewise do not determine how many reacted groups form load-bearing junctions. Diffusion/FRAP and scattering measurements provide complementary estimates of mesh or correlation length. Because these methods probe different physical quantities, agreement should not be assumed; multimodal characterization is preferable for heterogeneous or highly swollen networks (Richbourg et al., 2021; Lih et al., 2019).

Dynamic networks require an additional kinetic description. Relevant quantities include association and dissociation rate constants, equilibrium constants, bond lifetime, gelation time, and macroscopic stress-relaxation time. Reported kinetic windows span orders of magnitude: a recent synthesis of dynamic covalent bonds lists bond lifetimes from approximately 10^-4–10^4 s for imines, 10^4–10^6 s for hydrazones, 10^3–10^5 s for acylhydrazones, 10–10^2 s for boronate esters, and 10–10^3 s for thiol–disulfide exchange, depending strongly on substituents and solution conditions (Li et al., 2024). These values should therefore be treated as chemistry-dependent reference windows rather than universal material constants. Measurements should be performed at the intended temperature, pH, ionic strength, and competing-nucleophile concentration, because physiological conditions can shift both equilibrium and exchange kinetics.

Representative quantitative descriptors and their interpretation limits are collected in Table 2.

**TABLE 2:** Representative quantitative windows and how they should be interpreted.

Descriptor	Representative quantitative window/example	Interpretation/caveat	Recommended reporting
Dynamic-bond lifetime τB	Imine ∼10^-4–10^4 s; hydrazone ∼10^4–10^6 s; acylhydrazone ∼10^3–10^5 s; boronate ester ∼10–10^2 s; thiol–disulfide ∼10–10^3 s	Chemistry-level ranges span orders of magnitude and depend on substituent, pH, ionic strength and catalyst; not universal hydrogel constants	Temperature, pH, ionic strength, K_eq, k_on/k_off where available, and macroscopic relaxation time (Li et al., 2024)
Crosslink density/mesh size	No universal biopolymer range is defensible across formulations	Swelling, rheology, spectroscopy, scattering and diffusion probe different quantities; network defects violate ideal assumptions	At least two orthogonal methods; specify model assumptions and hydrated state (Richbourg et al., 2021; Lih et al., 2019)
Transport example	Alginate-resveratrol: 24-h release 54%/52% at pH 6.8/7.4; alginate/GNP/resveratrol 88%/66%	Same Ca2+-gelation trigger but secondary phase changes matrix-cargo transport	pH, cargo loading, geometry, release medium and secondary-phase loading (Mormile et al., 2025)
Bone composite example	ADA-GEL/PDA: contact angle 26° vs. 58°; 24-h fluid uptake 2648% ± 173% vs. 1635% ± 639%; compressive strength 0.69 ± 0.02 MPa	Illustrates simultaneous changes in interface, swelling and mechanics; cannot attribute biology to crosslinking alone	Matched uncoated scaffold plus surface/interfacial characterization (Ghorbani et al., 2022)
Bioprinting evidence	Performance should be reported as a coupled process window, not a single modulus	Print fidelity, rheology and cell stress are formulation- and platform-dependent	Yield stress/recovery, curing dose, filament fidelity, viability and maturation (Rajalekshmi and Agrawal, 2026)

### 4.2 Collagen and gelatin

Collagen offers native cell-recognition motifs and fibrillar organization, but its structure is sensitive to extraction, temperature, pH, ionic conditions, and crosslinking. Gelatin sacrifices native triple-helical order but gains solubility and chemical accessibility. These differences explain why the two materials respond differently to the same stabilization strategy. In collagen, preserving fibrillar architecture and protease-mediated remodeling may be as important as increasing modulus. In gelatin and GelMA, processing and crosslinking can be used more aggressively to build printed or patterned constructs, but cell behavior remains dependent on residual ligand accessibility and degradation kinetics. GelMA has become especially useful for decoupling architecture from bulk composition. Three-dimensional GelMA environments have been used to improve maturation of human iPSC-derived cardiomyocytes compared with two-dimensional conditions, emphasizing the combined importance of matrix architecture and co-culture context (Gisone et al., 2025). More recently, silk-fibroin reinforcement was introduced into GelMA to generate compartmentalized kidney-inspired hydrogels with tunable stiffness, swelling, degradation, and region-specific cell organization (Natarajan et al., 2026). The lesson is not that one composite is universally optimal, but that crosslinking can be spatially programmed to reproduce tissue heterogeneity.

### 4.3 Hyaluronic acid

HA is highly hydrated and biologically relevant to many soft tissues, but native HA lacks sufficient mechanical persistence for most scaffold applications. Chemical modification therefore becomes inseparable from function. Methacrylation enables photocrosslinking; aldehyde, hydrazide, catechol, or thiol functionalities enable dynamic or adhesive networks; and HA can be combined with gelatin, collagen, or chitosan to increase cell adhesion and structural stability (Burdick and Prestwich, 2011; Highley et al., 2016). The critical issue is to preserve the biological advantages of HA while avoiding a network that is either too labile or too resistant to remodeling. Injectable chitosan/HA hydrogels with aldehyde and catechol functionalities provide an instructive example: gelation, degradation, tissue adhesion, and stem-cell retention were co-optimized for cartilage-oriented delivery rather than treated as independent endpoints (Lin et al., 2025). HA thus illustrates why crosslinking should be viewed as a multi-objective optimization problem.

### 4.4 Silk fibroin

SF spans a broad range of formats because its beta-sheet content, molecular weight, and processing conditions can be tuned to produce films, fibers, sponges, hydrogels, and printed constructs (Vepari and Kaplan, 2007; Rockwood et al., 2011; Mushtaq et al., 2025). The main crosslinking question is how to gain sufficient shape and mechanical stability without locking the protein into a slowly degrading state that prevents cell infiltration or tissue replacement (Mushtaq et al., 2026). Recent work underscores the importance of degradation programming. Zhu et al. (2025) showed that the degradation behavior of SF hydrogels changes the proliferation and chondrogenic behavior of encapsulated stem cells. Methacrylated SF-based composite hydrogels have also been used to couple ROS scavenging with osteogenic activity in diabetic bone defects (Wang et al., 2025), while SF-based bioinks supplemented with decellularized ECM and gelatin have been developed to support 3D bioprinting for meniscus engineering (Fritz et al., 2025). Together these studies move SF design away from static “mechanical reinforcement” toward coordinated control of degradation, microenvironmental stress, and tissue-specific architecture.

### 4.5 Chitosan, alginate, fibrin, and related polysaccharide/protein matrices

Chitosan is cationic, chemically modifiable, and compatible with films, fibers, sponges, particles, and hydrogels. Its performance is strongly affected by molecular weight and degree of deacetylation, which should be reported because both influence solubility, charge density, degradation, and cell interaction (Dash et al., 2011; Pramanik et al., 2024). Crosslinking can improve wet-state mechanics but can also reduce the availability of protonated amino groups that contribute to adhesion and bioactivity. HA/chitosan combinations are therefore useful because they integrate electrostatic, covalent, and adhesive interactions within a single polysaccharide-rich network (Lin et al., 2025). Alginate provides rapid ionic gelation and excellent processability but comparatively limited intrinsic cell adhesion. It is therefore an ideal example of a material whose biological performance depends on architecture, functionalization, and added bioactive cues rather than polymer identity alone (Lee and Mooney, 2012). Recent PRP-loaded meniscal hydrogels and injectable tendon-regeneration gels demonstrate that calcium-crosslinked alginate can support meaningful *in vivo* repair when combined with appropriate biological and mechanical context (Resmi et al., 2025; Yamaguchi et al., 2025). Fibrin represents the opposite design extreme: it is highly bioactive and enzymatically remodeled, but its weak and rapidly evolving mechanics often require blending or secondary stabilization. Across these matrices, the goal is not maximal persistence but synchronization between network loss and tissue gain. These matrix-specific crosslinking trade-offs and the corresponding mechanistic validation priorities are summarized in Table 3.

**TABLE 3:** Matrix-specific crosslinking considerations in regenerative biopolymer biomaterials.

Matrix	Common routes	Primary benefit	Main risk	Validation priority
Collagen	EDC/NHS, genipin, enzymatic, fibrillogenesis	ECM-like architecture + wet stability	Ligand masking; reduced remodeling	Fibril structure, wet mechanics, enzymatic degradation, cell-mediated remodeling
Gelatin/GelMA	Methacrylation + photopolymerization; enzymatic	Spatial/temporal control; printability	Radical/light exposure; gradients	Degree of substitution, light dose, modulus + relaxation, diffusion, spreading
Hyaluronic acid	Methacrylation, hydrazone, Schiff-base, thiol, catechol	Injectability, dynamics, hydration	Rapid degradation or over-functionalization	Exchange kinetics, degradation, adhesion, immune response
Silk fibroin	Beta-sheet induction, enzymatic, methacrylation	Tunable mechanics/degradation	Excess beta-sheet slows remodeling	Secondary structure, degradation kinetics, encapsulated-cell behavior
Chitosan	Ionic, genipin, Schiff-base, catechol	Adhesion/cationic biointerface	Loss of amino groups; pH dependence	Deacetylation, charge, wet adhesion, degradation products
Alginate	Ca2+ ionic, covalent modification, IPN	Rapid mild gelation, printing	Ion exchange; poor intrinsic adhesion	Ca2+ distribution, swelling, relaxation, ligand functionalization
Fibrin	Thrombin-mediated, blending/secondary crosslinking	Native bioactivity/remodeling	Weak mechanics; rapid contraction	Clot architecture, contraction, proteolysis, integration

### 5 Processing and crosslinking are inseparable

Processing changes the state in which crosslinking occurs and therefore alters the final network. Figure 2 summarizes this coupling. A pre-crosslinked material may have high viscosity but poor cell-mixing homogeneity; *in situ* crosslinking may preserve injectability but expose cells to radicals, light, ionic gradients, or reactive groups; post-processing crosslinking may stabilize geometry but create gradients through limited diffusion or light penetration. The same nominal formulation can therefore produce different microenvironments depending on when and how the network is formed.

**FIGURE 2:**
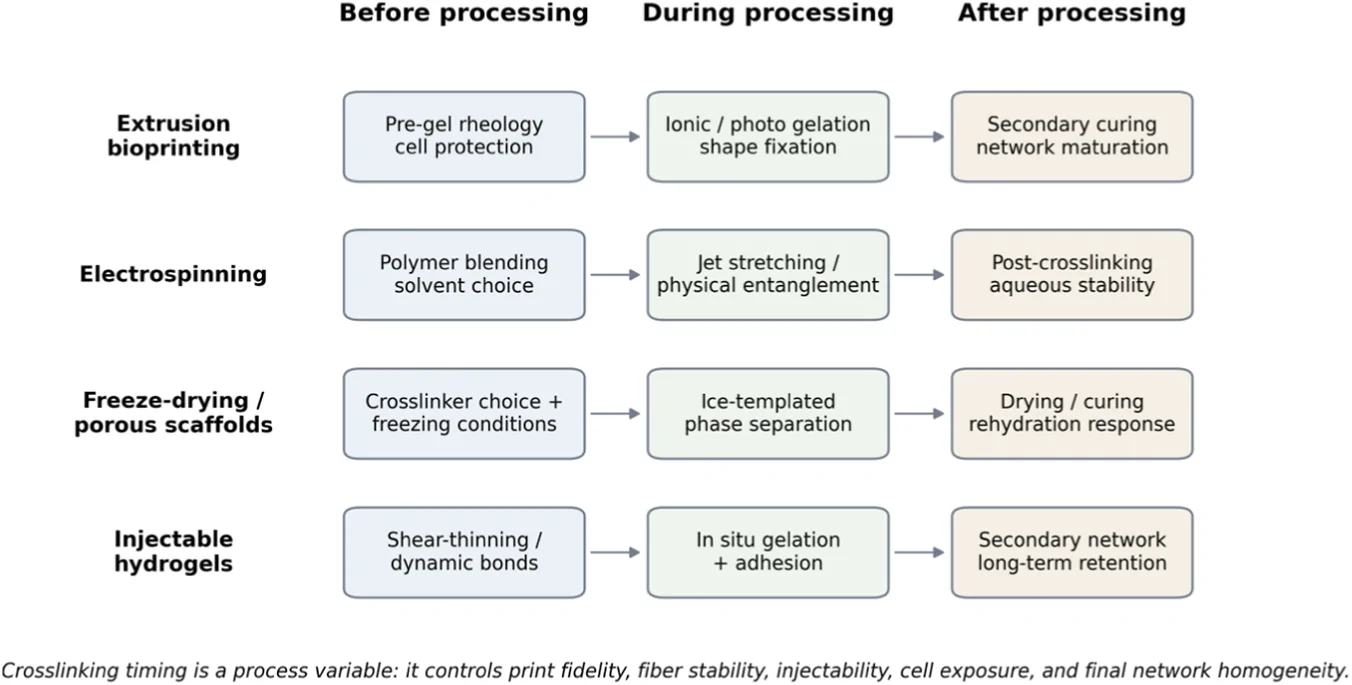
Processing–crosslinking timing map. The same polymer formulation can yield different hydrated networks depending on whether crosslinking occurs before, during, or after extrusion bioprinting, electrospinning, freeze-drying, or injection. Original schematic prepared for this Review.

### 5.1 Extrusion bioprinting and bioinks

Bioinks require a difficult compromise between printability and post-print biological function. Shear-thinning improves extrusion, rapid recovery supports filament fidelity, and crosslinking after deposition preserves geometry, but each strategy changes the stresses experienced by encapsulated cells. Definitions proposed for bioinks emphasize that material properties must be considered together with cell compatibility and printing performance (Groll et al., 2018; Gungor-Ozkerim et al., 2018). Recent formulations illustrate the need for coupled optimization. Alginate/cellulose bioinks loaded with thrombocyte concentrate were designed to maintain printability while introducing regenerative bioactivity (Grandjean et al., 2024). GelMA/nanohydroxyapatite systems have been modified to improve extrusion fidelity without allowing calcium release from the mineral phase to compromise GelMA gelation (Codrea et al., 2024). In SF-containing systems, addition of decellularized ECM and gelatin improved the balance between printability and meniscus-relevant biological support (Fritz et al., 2025). These examples show why print fidelity should be reported together with cell viability, matrix maturation, and hydrated-state mechanics.

### 5.2 Electrospinning and fibrous constructs

Electrospinning generates high-surface-area fibrous architectures that can mimic aspects of ECM topology and guide cell alignment (Pham et al., 2006; Agarwal et al., 2008; Bhardwaj and Kundu, 2010). For water-soluble or weakly stabilized biopolymers, however, the fiber morphology observed in the dry state may not survive hydration. Post-spinning crosslinking is therefore frequently required, and the resulting chemistry can modify fiber diameter, fusion, porosity, surface ligand accessibility, and degradation. A rigorous study should characterize the scaffold after crosslinking and rehydration rather than infer biological architecture from as-spun images.

### 5.3 Freeze-drying, porous scaffolds, and injectable hydrogels

Freeze-drying creates interconnected pores through ice templating, but pore size and anisotropy depend on polymer concentration, freezing rate, thermal gradients, and whether crosslinking occurs before or after drying. These variables affect rehydration, wet mechanics, and cell infiltration. Injectable hydrogels face a different constraint: the network must form quickly enough to remain localized but slowly enough to permit mixing and minimally invasive delivery. Dynamic covalent networks, ionic gels, and dual-network strategies are increasingly used to navigate this window (Guo et al., 2024; Lin et al., 2025). The coupling between processing route, crosslinking timing, material outputs, biological risks, and recommended controls is summarized in Table 4.

**TABLE 4:** Processing–crosslinking coupling and recommended controls.

Route	Timing issue	Key outputs	Biological risk	Recommended control
Extrusion bioprinting	Pre-, in-, or post-print gelation	Yield stress, recovery, fidelity, homogeneity	Shear damage; nonuniform curing	Cast control; matched curing dose; post-print viability
Electrospinning	Post-crosslinking often needed	Hydrated fiber diameter/orientation, porosity, tensile response	Residues; fusion; altered ligand presentation	As-spun vs. crosslinked vs. hydrated
Freeze-drying	Crosslink before/after freezing	Pore size/anisotropy, rehydration, wet compression	Dry morphology may misrepresent cell environment	Hydrated morphology/mechanics; controlled freezing history
Injectable hydrogel	Gelation must balance mixing/localization	Gel time, shear-thinning, recovery, adhesion	Premature gelation; cytotoxic groups	Injection-simulated rheology; material-only/crosslinker controls
Molded/patterned	Light/reagent penetration gradients	Spatial stiffness, conversion, swelling/degradation	Regional behavior from unintended gradients	Depth-resolved characterization

## 6 From network properties to biological mechanism

### 6.1 Stiffness, viscoelasticity, and mechanotransduction

The widespread use of elastic modulus as a biomaterial descriptor has been productive but can also be misleading. Cells apply forces over time, and many biopolymer networks relax, reorganize, or degrade during that process. Engler et al. (2006) established that matrix elasticity can influence lineage specification, while Chaudhuri et al. (2016) demonstrated that stress-relaxation behavior independently affects stem-cell fate and activity. For crosslinked biopolymers, the practical implication is that two hydrogels with similar small-strain modulus can produce different biological responses if one permits rapid stress dissipation or local remodeling. This distinction is especially relevant to dynamic and physically crosslinked networks. A material that is initially soft but unable to relax may constrain spreading more strongly than a nominally stiffer matrix that reorganizes under cell traction. Mechanical characterization should therefore include, when relevant, stress relaxation, frequency-dependent rheology, cyclic loading, or creep rather than a single modulus measured at one time point. Tissue-specific mechanical targets should be treated as ranges and time-dependent behaviors, not as one scalar number.

### 6.2 Swelling, transport, and degradation

Crosslinking simultaneously governs molecular transport and degradation. Increasing network density typically reduces swelling and mesh size, which can slow nutrient, protein, growth-factor, and drug diffusion. The same change may reduce enzymatic accessibility and extend scaffold persistence. These effects can be beneficial in one context and harmful in another: a barrier membrane may require sustained integrity, whereas a cell-instructive hydrogel may require rapid matrix replacement. Degradation studies are most informative when linked to the mechanism of network loss. Mass loss alone cannot distinguish hydrolysis, enzymatic cleavage, ionic exchange, chain scission, or dissolution. Moreover, degradation products can themselves alter local osmolarity, pH, immune response, or cell viability. SF work linking hydrogel degradation to chondrogenesis provides a strong example of treating degradation as a biological variable (Zhu et al., 2025). Similar logic applies to alginate and HA systems, in which ion exchange or dynamic-bond exchange can change mechanics before substantial mass is lost. An experimentally useful transport benchmark is provided by Mormile et al. (2025), who formed alginate-resveratrol and alginate/GNP/resveratrol beads by the same Ca2+-mediated ionic gelation route. Although developed for controlled delivery rather than tissue repair, the system isolates an important crosslinking principle: an identical biopolymer network-forming trigger can yield different transport behavior when a secondary phase changes matrix-cargo interactions. After 24 h, alginate-resveratrol beads released 54% and 52% of the loaded resveratrol at pH 6.8 and 7.4, respectively, whereas the GNP-containing beads released 88% and 66%. Figure 3 summarizes this open-access experimental case as a crosslinking-to-transport example while keeping alginate as the continuous matrix.

**FIGURE 3:**
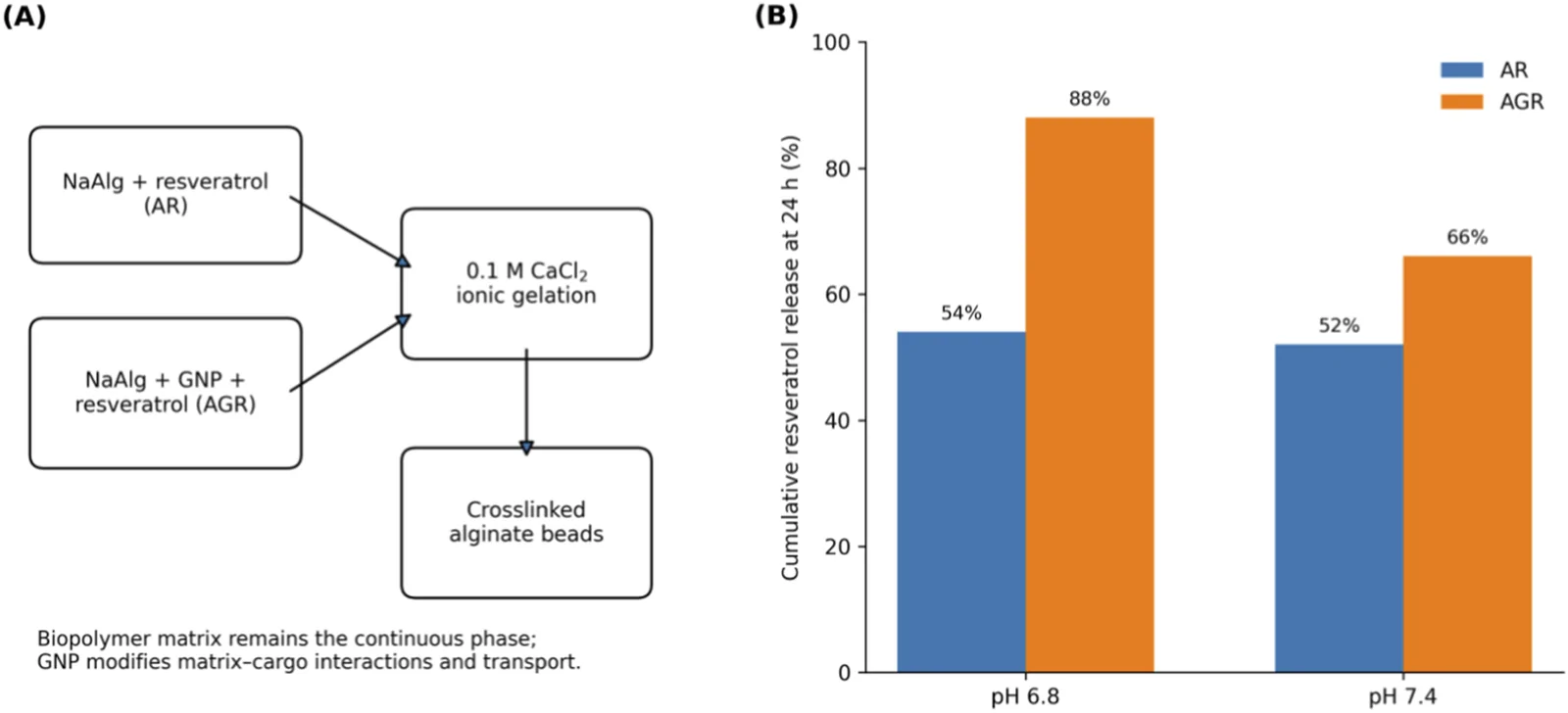
Literature-derived experimental example of ionic crosslinking and transport in an alginate matrix. **(A)** Author-redrawn preparation logic for alginate-resveratrol (AR) and alginate/GNP/resveratrol (AGR) beads formed by Ca2+-mediated ionic gelation. **(B)** Author-redrawn comparison of the 24 h cumulative resveratrol release reported for AR and AGR at pH 6.8 and 7.4. Data and preparation conditions are from Mormile et al. (2025), Gels 11, 15. The source article is distributed under the Creative Commons Attribution 4.0 International (CC BY 4.0) license. All artwork in this figure was newly prepared for the present Review from the cited source data and concepts; no published artwork is reproduced. Bellucci | Crosslinking-Controlled Biopolymer Biomaterials | Frontiers in Bioengineering and Biotechnology v i ew In re.

### 6.3 Ligand presentation, immune response, and matrix remodeling

Crosslinking can change the availability of cell-binding motifs by consuming functional groups, restricting chain mobility, or altering protein conformation. It can also modify protein adsorption and the composition of the provisional matrix that forms at the material surface. These changes influence macrophage phenotype, fibroblast activation, endothelialization, and foreign-body response (Anderson et al., 2008; Witherel et al., 2019). Consequently, a crosslinking strategy that improves mechanical stability but increases fibrotic encapsulation may reduce regenerative value. Immune-aware material design is therefore moving from descriptive cytokine measurement toward causal relationships between network state and host response. The dual-crosslinked HA system of Guo et al. (2024), for example, linked the material platform to macrophage M2 polarization and bone formation. Bioactive macromolecules can also be integrated into biopolymer constructs to provide regenerative signaling, but their contribution should be separated from the effect of crosslinking itself. Recent discussion of erythropoietin in tissue engineering illustrates how bioactive cues can influence angiogenic, immunomodulatory, and regenerative processes when incorporated into engineered microenvironments (Sanghvi et al., 2026).

## 7 Tissue-specific design windows

### 7.1 Bone and osteochondral regeneration

Bone-oriented biopolymer matrices usually require greater mechanical stability and mineralization support than soft-tissue scaffolds, but excessive stiffness or slow degradation can impair vascular invasion and replacement by new tissue. Mineral phases such as HAp or bioactive glass are therefore most useful when their loading is coupled to network mechanics, ion release, and cell response rather than treated as a simple filler addition (Rezwan et al., 2006; Filip et al., 2022). Recent studies provide multiple examples of this coupling. Strontium-doped HAp/GelMA scaffolds have been 3D printed to combine photopolymerized architecture with osteoconductive mineral cues (Codrea et al., 2024). GO/GelMA hydrogels were evaluated with both antibacterial and osteogenic endpoints in periodontal bone defects (Cheng et al., 2026). Methacrylated SF composites were designed to address ROS-rich diabetic bone environments while maintaining osteogenic capacity (Wang et al., 2025). In each case, the regenerative claim is strongest when matrix crosslinking, mineral or nanophase loading, and biological function are experimentally separated. A directly regenerative example is the 3D-printed alginate-dialdehyde/gelatin (ADA-GEL) platform reported by Ghorbani et al. (2022). In that study, oxidation-enabled covalent coupling between ADA and gelatin was combined with post-print CaCl2 crosslinking, followed by polydopamine (PDA) surface functionalization. The PDA-coated scaffolds showed a lower water contact angle (26° versus 58°), greater 24 h fluid uptake (2648% ± 173% versus 1635% ± 639%), a reported compressive strength of 0.69 ± 0.02 MPa, and MG-63 viability of 140.18% after 7 days, together with increased ALP activity and mineralization. Figure 4 uses these published data to illustrate how crosslink chemistry, processing sequence, surface functionalization, and biological validation can be connected within a single bone-regeneration platform. Osteochondral repair adds a gradient problem because cartilage and subchondral bone require different mechanics, transport, and degradation. Crosslinking gradients or compartmentalized constructs may therefore be more biologically appropriate than a uniform “average” stiffness. The same concept underlies compartmentalized SF/GelMA platforms used to reproduce region-dependent tissue architecture in other organs (Natarajan et al., 2026).

**FIGURE 4:**
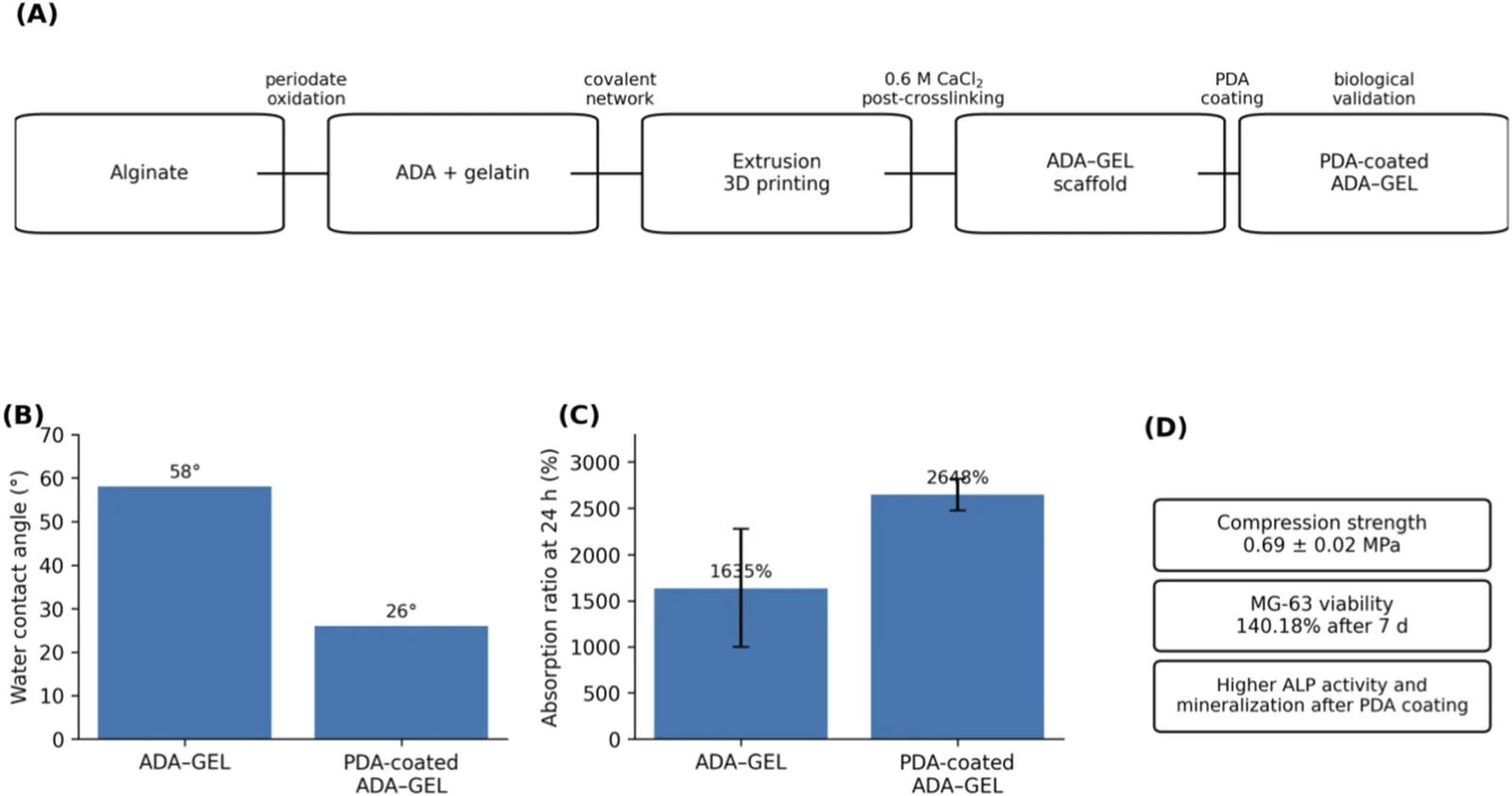
Literature-derived experimental example linking crosslinking sequence, processing, surface functionalization, and biological validation in a bone-regeneration scaffold. **(A)** Author-redrawn sequence from alginate oxidation to ADA-GEL formation, extrusion 3D printing, CaCl2 post-crosslinking, and PDA coating. **(B)** Water contact angle and **(C)** 24 h absorption ratio reported for ADA- GEL and PDA-coated ADA-GEL scaffolds. **(D)** Selected reported endpoints for the PDA-coated scaffold, including compression strength, MG-63 viability after 7 days, and increased ALP activity/mineralization. Data and design logic are from Ghorbani et al. (2022), Front. Bioeng. Biotechnol. 10, 940070, an open-access article distributed under the Creative Commons Attribution License (CC BY). All artwork in this figure was newly prepared for the present Review; no published microscopy or other original artwork is reproduced. Bellucci | Crosslinking-Controlled Biopolymer Biomaterials | Frontiers in Bioengineering and Biotechnology.

### 7.2 Skin and wound repair

Wound biomaterials must tolerate exudate, movement, oxidative stress, microbial challenge, and repeated mechanical deformation. Crosslinking therefore affects not only persistence but also adhesion, fluid uptake, permeability, and removal. Dynamic networks are attractive because they can self-heal or dissolve on demand, reducing tissue trauma during dressing changes (Johnson et al., 2024). Chitosan-containing systems add cationic and adhesive functionality, whereas gelatin, HA, and SF contribute hydrated matrices and cell-interactive motifs (Pramanik et al., 2024). A key design tension is that highly crosslinked wound dressings may resist dissolution but impede cell infiltration and tissue integration. Conversely, very labile hydrogels can lose barrier function before re-epithelialization. Time-resolved wet mechanics, swelling, degradation, antimicrobial performance, fibroblast/keratinocyte response, and *in vivo* closure or histological remodeling should therefore be assessed within the same formulation space.

### 7.3 Cartilage, tendon, nerve, vascular, and soft tissues

Cartilage engineering requires hydrated load-bearing matrices that support chondrogenic phenotype while resisting excessive swelling. Enzymatically crosslinked collagen II/HA systems and adhesive chitosan/HA hydrogels illustrate two different solutions: one prioritizes biomimetic cartilage composition, the other injectable retention and stem-cell delivery (Torabi Rahvar et al., 2024; Lin et al., 2025). Meniscal repair similarly benefits from injectable alginate matrices when biological cues and *in vivo* remodeling are evaluated together (Resmi et al., 2025). Tendon and nerve applications highlight anisotropy and mechanical guidance. The injectable alginate–cell cross-linked strategy reported by Yamaguchi et al. (2025) used cell orientation as part of the regenerative mechanism rather than simply as a histological endpoint. In neural-oriented systems, antioxidant GelMA composites have been developed to protect Schwann cells under oxidative stress (Han et al., 2025). Vascular and cardiac constructs require yet another balance: compliance, cell alignment, endothelial compatibility, and long-term dynamic loading are more relevant than high static modulus. Three-dimensional GelMA environments that improve cardiomyocyte maturation emphasize the importance of matrix context beyond nominal polymer composition (Gisone et al., 2025).

## 8 Mechanistically informed biological validation

A central weakness in the biomaterials literature is the frequent use of biological assays as confirmation rather than as mechanistic tests. Cell viability is necessary but rarely sufficient. Figure 5 proposes an evidence chain in which each biological conclusion is linked backward to a defined material property. If a stiffer scaffold increases osteogenic markers, for example, the study should ask whether stiffness changed independently of porosity, mineral content, degradation, protein adsorption, or cell density. Without such controls, attribution to mechanotransduction remains uncertain. *In vitro* studies should include material-only controls, crosslink-density or crosslinker controls, relevant uncrosslinked or alternative-network comparators, and time-resolved measurements. Three-dimensional culture is preferable when the claim concerns cell–matrix interactions within the scaffold. For hybrid biomaterials, dose-response studies and leachate testing are important because soluble degradation products or released ions can drive biological effects independently of the bulk matrix. *Ex vivo* models can bridge simplified cell culture and whole-animal studies by preserving tissue architecture, matrix composition, and resident cell populations. They are particularly useful for skin, cartilage, vascular, nerve, and osteochondral interfaces, although diffusion limits and limited observation windows must be acknowledged. *In vivo* studies should be selected to test the specific regenerative mechanism claimed by the material. Histology alone is not enough when functional recovery, mechanical integration, or degradation matching is central to the hypothesis. Recent studies illustrate stronger validation logic. PRP-loaded alginate hydrogels were compared with sham and hydrogel-only controls in a rabbit meniscal model (Resmi et al., 2025). GO/GelMA periodontal constructs were tested through antibacterial and osteogenic assays before *in vivo* defect evaluation (Cheng et al., 2026). The dual-network HA system linked mechanical and immune-regulatory behavior to rat calvarial repair (Guo et al., 2024). Such multi-level evidence is more persuasive than a large number of unrelated endpoints because it establishes a traceable path from network design to regenerative outcome.

**FIGURE 5:**
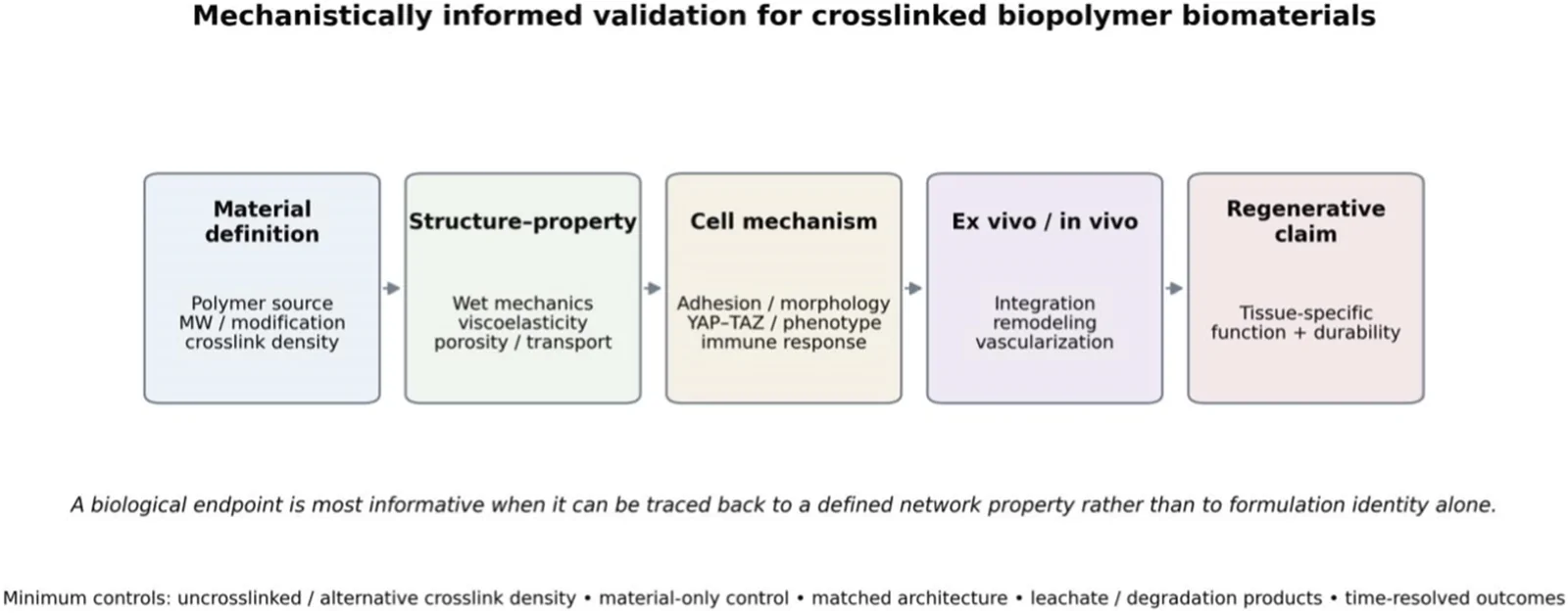
Mechanistically informed validation chain for crosslinked biopolymer biomaterials. Strong regenerative claims require a traceable sequence from material definition and hydrated-state properties to cellular mechanism, *ex vivo*/*in vivo* integration, and tissue-specific function. Original schematic prepared for this Review.

## 9 Reproducibility, sterilization, storage, and aging

Crosslinked biopolymer biomaterials are unusually sensitive to manufacturing history because natural polymers vary in source, molecular weight, degree of substitution, impurity burden, and supramolecular organization. Batch-to-batch variability can therefore change gelation kinetics and mechanics even when nominal concentrations are identical. These parameters should be reported explicitly and, when possible, linked to acceptance ranges. Sterilization is another hidden crosslinking variable. Gamma irradiation and electron beams can cause chain scission or additional crosslinking depending on polymer chemistry and hydration state; ethylene oxide can leave residues; UV exposure can alter chromophores and photoreactive groups; and filtration is not possible for many assembled constructs (Galante et al., 2018). Storage can produce oxidation, hydrolysis, crystallization, physical aging, or freeze–thaw damage. Figure 6 summarizes these sources of property drift. A useful minimum standard is to measure key network properties after the actual sterilization and storage protocol intended for use, not only in the freshly prepared laboratory material. The same principle applies to scale-up. Mixing intensity, oxygen exposure, temperature gradients, light penetration, and diffusion distances change with batch size. A photopolymerized construct that is homogeneous at millimeter scale may develop crosslink gradients in a thicker geometry. A freeze-dried scaffold can develop different pore anisotropy when cooling rates change. Translational robustness therefore requires manufacturing controls to be integrated into the design phase rather than added after biological optimization.

**FIGURE 6:**
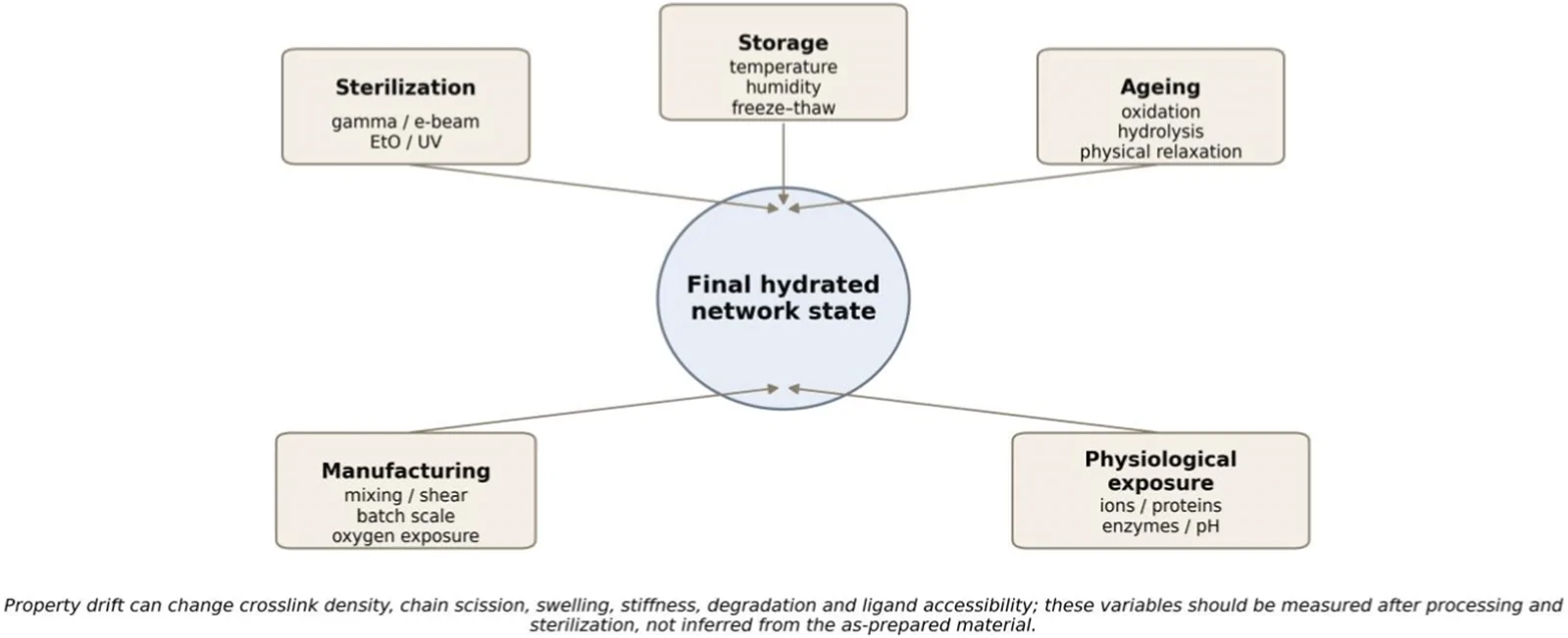
Hidden variables that can modify the final crosslinked network. Sterilization, storage, physical aging, manufacturing scale, and physiological exposure can cause chain scission, additional crosslinking, hydrolysis, oxidation, ionic exchange, or physical relaxation. Key material properties should therefore be measured after relevant processing and storage steps. Original schematic prepared for this Review.

## 10 Discussion: controversies, gaps, and design priorities

The first unresolved issue is the tendency to equate greater crosslinking with better biomaterial performance. Stability is beneficial only until it begins to suppress transport, remodeling, or integration. For regenerative matrices, the useful objective is not maximal modulus or minimal degradation but a time-dependent design window matched to the target tissue. This is particularly clear in dynamic and dual-network hydrogels, where reversible bonds can provide injectability and stress relaxation while a secondary network preserves long-term shape (Guo et al., 2024). A second controversy concerns the interpretation of stiffness. Static elastic modulus remains widely reported because it is simple and comparable, but many biopolymer matrices are viscoelastic, poroelastic, and degrading. The field should therefore move toward mechanical descriptors that reflect the time scales of cell traction and tissue loading. Stress relaxation, cyclic recovery, creep, fatigue, and evolution during degradation may be more biologically informative than one initial modulus. The mechanobiology literature already demonstrates that time-dependent mechanics can alter stem-cell behavior independently of initial stiffness (Chaudhuri et al., 2016). A third gap is the weak separation between formulation complexity and mechanism. Hybrid systems often add mineral particles, graphene-related materials, metal oxides, growth factors, exosomes, or other bioactive agents. Such additions may improve a headline endpoint while simultaneously changing crosslinking, porosity, surface area, degradation, or protein adsorption. The biopolymer matrix should remain experimentally interpretable. Matched polymer-only controls, filler dose-response, leaching analysis, and characterization of the hydrated composite are therefore essential. This point is especially important for Research Topic contributions in which hybrid components are present but the biopolymer is intended to remain the central regenerative platform. A fourth gap is reporting discipline. Polymer source and molecular weight, degree of modification, crosslinker-to-functional-group ratio, curing dose, gel fraction, swelling, wet-state mechanics, degradation conditions, and sterilization history are not consistently reported. The absence of these variables makes apparently similar studies difficult to compare. Table 5 proposes a minimum evidence set designed to improve reproducibility without requiring every study to perform every possible assay. Network heterogeneity is a related but underreported variable. Crosslinking reactions occur in three-dimensional space and can be limited by light attenuation, reagent diffusion, oxygen inhibition, local water content, or phase separation. Consequently, a construct can have the same average modulus as another sample while containing very different local stiffness, mesh-size, or degradation gradients. These gradients may be intentional, as in osteochondral or compartmentalized tissues, or accidental consequences of manufacturing. The distinction should be made experimentally. Spatial mapping of mechanics, conversion, swelling, or degradation is particularly valuable for thick printed constructs and multiphase hydrogels, because bulk measurements can conceal regions that cells experience as mechanically or chemically distinct microenvironments. This also argues for reporting sample dimensions and curing geometry together with nominal crosslinking conditions. Finally, biological validation should become more mechanistic and more tissue-specific. High cell viability on a scaffold is not evidence that the material actively guides regeneration. Stronger claims require evidence that a defined material property changes cell behavior, host response, matrix deposition, vascularization, organization, or function in a manner relevant to the target tissue. Recent primary studies in meniscus, tendon, bone, nerve, and multicompartment organ models show that this standard is increasingly achievable (Han et al., 2025; Resmi et al., 2025; Yamaguchi et al., 2025; Cheng et al., 2026; Natarajan et al., 2026).

**TABLE 5:** Mechanistic evidence matrix linking network chemistry to measurable properties and biological outcomes.

Crosslinking chemistry/claim	Measurable network property	Biological mechanism	Tissue-specific outcome/evidence	Critical control
Permanent/photo-covalent; increased stability	Conversion, effective crosslink density, wet modulus, mesh size	Traction resistance, ligand accessibility, transport	Shape retention; bone/skin/cartilage support	Matched polymer concentration and architecture
Dynamic covalent; improved remodeling	Stress-relaxation time, k_on/k_off, K_eq, bond lifetime	Cell spreading, migration, matrix deposition	Injectable/self-healing soft-tissue and bone matrices	Static network with similar initial modulus
Ionic/physical; reversible stabilization	Ion distribution, exchange rate, relaxation, swelling	Force dissipation, ion-mediated drift, remodeling	Alginate meniscus/tendon; SF remodeling	Matched ionic environment and ligand density
Hybrid phase provides benefit	Interfacial state, dispersion, leaching/release, network perturbation	Adsorption, ion release, redox/immune signaling	Bone, periodontal, nerve and wound models	Matrix-only, secondary-phase, dose-response controls
Preclinical robustness	Post-sterilization chemistry, mechanics, degradation, storage stability	Preservation of cell-material cues over shelf/use life	Reproducible *in vitro*/*ex vivo*/*in vivo* performance	Test actual sterilized/stored configuration

## 11 Future directions

Several directions are likely to define the next phase of crosslinked biopolymer biomaterials. Dynamic covalent and supramolecular networks will be used increasingly to reproduce time-dependent ECM behavior, but their bond-exchange kinetics must be measured under physiological conditions rather than inferred from chemistry alone. Spatially patterned crosslinking will enable gradients and compartments that better reproduce tissue heterogeneity. Non-destructive monitoring, including optical approaches sensitive to network state, may allow aging, curing, and degradation to be followed without sacrificing samples (Rodriguez and Quinn, 2026). Biofabrication will also shift from geometry-first optimization toward coupled material–cell process windows. Printing parameters, crosslink timing, cell stress, architecture, and post-print maturation should be optimized together. Data-driven methods may help navigate this multidimensional space (Zhang et al., 2025), but meaningful machine-learning models will require standardized input descriptors and biological endpoints. A model cannot compensate for missing information about polymer molecular weight, crosslink density, or sterilization history. Most importantly, the field should define success in terms of controlled evolution. Regenerative biomaterials are temporary microenvironments. Their crosslinks should preserve the construct when needed, relax when cells need to generate force, permit transport when tissue metabolism demands it, and disappear as new matrix becomes functional. Designing that sequence requires integration of polymer chemistry, mechanics, degradation, processing, immunology, and tissue-specific biology.

## 12 Conclusion

Crosslinking is the central variable that converts a biopolymer formulation into a regenerative microenvironment. It determines much more than scaffold stability: it controls hydrated mechanics, stress relaxation, swelling, transport, degradation, ligand presentation, architecture, and the way cells and host tissues interact with the material. The most informative studies therefore connect crosslinking chemistry and processing to measured network properties and then to mechanistically relevant biological outcomes. Across collagen/gelatin, HA, SF, chitosan, alginate, fibrin, and related systems, no single crosslinking strategy is universally superior. Covalent networks provide persistence, physical and ionic networks provide mild and often reversible stabilization, dynamic covalent systems permit remodeling and injectability, and photo-crosslinking enables spatial and temporal control. The correct strategy depends on the target tissue and on the time-dependent balance among mechanical support, transport, degradation, and remodeling. Progress toward reproducible preclinical translation will depend on better reporting of polymer and network variables, characterization after sterilization and storage, matched experimental controls, and validation that traces biological outcomes back to defined material properties. When these requirements are met, crosslinked biopolymer biomaterials can move beyond passive scaffolds toward instructive, adaptive platforms that actively coordinate tissue regeneration.
